# Supplementary material for: Novel computational method for predicting polytherapy switching strategies to overcome tumor heterogeneity and evolution
Source: Sci Rep. 2017 Mar 13;7:44206. doi: 10.1038/srep44206 (PMC5347024; doi:10.1038/srep44206)
Supplement: Supplementary Information [file srep44206-s1.pdf]

Supplementary Information
Novel computational method for predicting polytherapy switching strategies to overcome tumor heterogeneity and evolution

Vanessa D. Jonsson[1,†], Collin M. Blakely[2,3,†], Luping Lin[2,3], Saurabh Asthana[2,3], Nikolai Matni[1], Victor Olivas[2,3], Evangelos Pazarentzos[2,3], Matthew A. Gubens[2,3], Boris C. Bastian[3,4], Barry S. Taylor[5,6,7], John C. Doyle[8,9,10], and Trever G. Bivona[2,3,11,*]

[1]Department of Computing and Mathematical Sciences, California Institute of Technology, Pasadena, CA
[2]Department of Medicine, University of California, San Francisco, CA
[3]Helen Diller Family Comprehensive Cancer Center, University of California San Francisco, San Francisco, CA
[4]Department of Pathology, University of California, San Francisco, CA
[5]Human Oncology and Pathogenesis Program, Memorial Sloan Kettering Cancer Center
[6]Department of Epidemiology and Biostatistics, Memorial Sloan Kettering Cancer Center
[7]Marie-Josée and Henry R. Kravis Center for Molecular Oncology, Memorial Sloan Kettering Cancer Center, New York, NY
[8]Department of Control and Dynamical Systems, California Institute of Technology, Pasadena, CA
[9]Department of Electrical Engineering, California Institute of Technology, Pasadena, CA
[10]Department of Biological Engineering, California Institute of Technology, Pasadena, CA
[11]Cellular and Molecular Pharmacology, University of California, San Francisco, CA
[†]co-first authors
[*]corresponding author

Corresponding author: Trever Bivona MD PhD (email: trever.bivona@ucsf.edu)

1 Mathematical Methods

1.1 Evolutionary Dynamics Model of NSCLC

The quasispecies model [1] was originally developed to describe the dynamics of populations of self replicating macromolecules undergoing mutation and selection. We choose this model for its relative simplicity and its ability to capture the salient features of the evolutionary dynamics of a simplified generic disease model. The following adaptation incorporates the effects of small molecule inhibitors and describes the growth, mutation and evolution of non small cell lung adenocarcinoma populations:

$$\dot{x}_i = r_i q_{ii} x_i + \sum_{k \neq i}^{n} r_i q_{ik} x_k - \Psi_i(\ell_k) x_i \tag{S1}$$

where $x_i \in \mathbb{R}_+$ is the concentration of a NSCLC subpopulation i, $\ell_k \in \mathbb{R}_+$ is a small molecule inhibitor concentration (assumed to remain at constant concentrations throughout), r_i is the growth rate for each cell x_i, and q_{ik} is the probability that cell k mutates to cell i (note that q_{ii} is the probability of no mutation occurring). Finally, the function $\Psi_i(\ell_k)$ represents the pharmacodynamics of individual drugs ℓ_k or of individual EGFR TKIs (erlotinib or afatinib) in combination with fixed concentrations other small molecule inhibitors used in this study (0.5 μM crizotinib, 0.5 μM trametinib or 5 μM vemurafenib) with respect to the i-th NSCLC cell type, namely:

$$\Psi_i(\ell_k) = \gamma_{ik} \frac{[\ell_k]^{n_{ik}}}{[\ell_k]^{n_{ik}} + K_{ik}^{n_{ik}}} \tag{S2}$$

where $\ell_k \in \mathbb{R}_+$ is the drug concentration, $\gamma_{ik} \in \mathbb{R}_+$ is the saturation coefficient, $K_{ik} \in \mathbb{R}_+$ is the dissociation constant, $n_k \in \mathbb{R}_+$ is the Hill coefficient. Equation S2 has previously been described in [2, 3]. When $\ell_k = 0$, $\forall k \in \{1, ..., m\}$, the dynamics are unstable.

2 A control theoretic algorithm for designing treatment strategies

To design treatment strategies that best minimize tumor size and control its evolution over time, we combine both a greedy algorithm and receding horizon control approach. We introduce some notation, cost function definitions and specify our algorithm.

2.1 Cost functions

To measure the effectiveness of a given treatment strategy over time, we define the *average cost* function. For a given treatment strategy ℓ_k applied to Equation (S1), we rewrite the dynamics of the entire system (i.e., for all cells) as

$$\dot{x} = A(\ell_k) x, \tag{S3}$$

where $A \in \mathbb{R}^{n \times n}$ is a matrix that represents the growth, mutation and drug dynamics for treatment strategy ℓ_k, for n cell subpopulations.

The *average cost* C_r for a time horizon N, allowable switching period τ and time intervals of the form $[k\tau, (k+1)\tau]$ for $k = \{0, .., N/\tau - 1\}$ is given by

$$C_r = \sum_{k=0}^{N/\tau - 1} \int_{k\tau}^{(k+1)\tau} \mathbf{1}^T x(t) dt \tag{S4}$$

where $\mathbf{1}^T$ is the $n \times 1$-dimensional vector of ones and $x(t)$ is the solution to Equation (S3).

Equation (S4) simplifies to

$$C_r = \sum_{k=0}^{N/\tau - 1} \mathbf{1}^T A^{-1} (e^{A_{\ell_k}((k+1)\tau - k\tau)} - I) x(k\tau). \tag{S5}$$

The *final cost* C_f for an inital tumor population $x(0)$ and a sequence of drugs $\{\ell(k)\}_{k=1}^{N/\tau-1}$ that define a switching therapy over a time horizon N is defined as

$$C_f = e^{A(\{\ell(k)\}_{k=1}^{N/\tau-1})}x(0). \tag{S6}$$

2.2 Algorithm

Our algorithm is defined as follows. Given an initial tumor population, denoted by x_0, a time horizon N and an allowable switching period τ, we perform the following computations to determine a candidate treatment strategy:

Algorithm 1 Treatment strategy synthesis

1. **Initialization:** Set $k = 0$ and $x(0) = x_0$.
2. **Greedy approach:** For time interval $[k\tau, (k+1)\tau]$, compute $y((k+1)\tau) = e^{A(\ell_k)\tau}y(k\tau)$ for each possible treatment strategy ℓ_k.
3. **Update**: Set $\ell(k) = \arg\min_{\ell_k} \text{sum}(y(k+1)\tau)$, and set $x((k+1)\tau) = \min_{\ell_k} \text{sum}(y(k+1)\tau)$. Increment k: if $k = N$, proceed to step 4, otherwise return to step 2.
4. **Output**: A sequence of drugs $\{\ell(k)\}_{k=1}^{N/\tau-1}$ that define a switching therapy.

The resulting switching therapy $\{\ell(k)\}$ is then applied until the next biopsy can be taken, giving a new tumor cell population measurement, at which point the algorithm is repeated. In particular, it is important that the horizon N be chosen to be longer than expected periods between biopsies.

3 Model Implementation and Simulations

3.1 Derivation of dynamical system parameters

Growth and Mutation Rates. We model the growth of NSCLC cell population x_i by the following ordinary differential equation (ODE):

$$\dot{x}_i = r_i x_i, \tag{S7}$$

where r_i is the growth rate per day, and $\dot{x}_i$ denotes the derivative with respect to time of the tumor cell population x_i. Note that we assume that no mutations occur over the time-frame considered, allowing us to set $q_{ii} = 1$ and $q_{ij} = 0$ in the dynamic model (S1), resulting in (S7).

Given an initial population $x_i(0)$, the population $x_i(t)$ on day t can be obtained by solving ODE (S7), and is specified by the following expression

$$x_i(t) = x_i(0)e^{r_i t}. \tag{S8}$$

Given a set of N experimental data points $e_i(0), e_i(t_1), \ldots, e_i(t_N)$, we fit these points to an exponential function of the form (S8), with $x_i(0) = e_i(0)$ to obtain an experimentally derived value for the growth rate r_i of tumor cell population x_i.

We take the DNA mutation rate to be $1e^{-9}$ mutation/base pair/cell division. We assume that mutations occur unidirectionally from $\text{EGFR}^{\text{L858R}}$ parental cells to $\text{EGFR}^{\text{L858R,T790M}}$, $\text{EGFR}^{\text{L858R}}$, $\text{BRAF}^{\text{V600E}}$ or $\text{EGFR}^{\text{L858R,T790M}}$ $\text{BRAF}^{\text{V600E}}$, HGF-/+. For a NSCLC cell population with growth rate r_i, the corresponding doubling time td_i (cell division per day) is

$$td_i = \frac{ln(2)}{r_i}. \tag{S9}$$

The mutation rate in units of mutation/base pair/day for an NSCLC cell population with doubling time td_i is $1e^{-9} \cdot td_i{}^{-1}$. The rate of mutation to one particular base pair/day is then approximated by $1e^{-9} \cdot g_s^{-1} \cdot td_i{}^{-1}$, where $g_s = 3e^9$ is the size of the human genome in base pairs.

Drug Effect Rates and Hill Functions. We model the change in a tumor cell population x_i under a treatment j of concentration ℓ with the following ordinary differential equation (ODE):

$$\dot{x}_i = r_i x_i - f_i^j(\ell) x_i, \tag{S10}$$

where r_i is the growth rate per day derived in the previous section and $f_i^j(\ell)$ is a function mapping the treatment j at concentration ℓ to a drug effect rate per day. We again assume that no mutation occurs over the time-frame considered, allowing us to set the mutation rates $q_{ii} = 1$ and $q_{ij} = 0$ in the model (S1), resulting in (S10).

Similar to the previous section, given an initial population $x_i(0)$, the population $x_i(t)$ on day t can be obtained by solving ODE (S10), and is specified by the following expression

$$x_i(t) = x_i(0) e^{(r_i - f_i^j(\ell) t}. \tag{S11}$$

We model the map $f_i^j(\ell)$ as a modified function of the form

$$f_i^j(\ell) = \gamma_{j,i} \frac{\ell^{n_{j,i}}}{\ell^{n_{j,i}} + K_{j,i}^{n_{j,i}}}, \tag{S12}$$

where $\gamma_{j,i}$ $n_{j,i}$ and $K_{j,i}$ are the saturation parameter, Hill function coefficient and binding reaction dissociation constant for drug j applied to cell x_i.

Our goal is to obtain values for these three parameters using experimental data measuring cell viability under varying concentrations ℓ of drug j. In particular, given experimentally obtained data pairs of the form $\ell, y_{i,j,\ell}(1)$, where $y_{i,j,\ell}(1)$ is the ratio of the tumor cell population x_i treated with concentration ℓ of drug j at day 1 to the tumor cell population x_i treated with no drug at day 1. Letting x_i^ℓ denote the treated tumor population and x_{ctrl}^ℓ denote the untreated control tumor population, it follows that $y_{i,j,\ell}$ can be written as

$$y_{i,j,\ell}(1) = \frac{x_i^\ell(1)}{x_i^{\text{ctrl}}(1)} = \frac{x_i(0) e^{(r_i - f_i^j(\ell))}}{x_i(0) e^{r_i}} = e^{-f_i^j(\ell)}, \tag{S13}$$

where the first equality follows from the definition of $y_{i,j,\ell}(1)$, the second from applying equations (S11) and (S8) to $x_i^\ell(1)$ and $x_i^{\text{ctrl}}(1)$ respectively, and the third from canceling like terms. It follows that the experimentally derived values of $f_i^j(\ell)$ are given by

$$f_i^j(\ell) = -\ln(y_{i,j,\ell}). \tag{S14}$$

Solving this equation for each experimentally tested concentration ℓ, we obtain a set of points $\{\ell, f_i^j(\ell)\}$ that can be used to derive the parameters $\gamma_{j,i}$ $n_{j,i}$ and $K_{j,i}$ via curve fitting. In order to avoid overfitting, we set $\gamma_{j,i} = \max_\ell f_i^j(\ell)$, i.e., we force the modified Hill function to saturate at the maximal experimentally observed rate. Although this approach can be conservative in modeling the drug effect rate of high concentrations of drugs, we note that the the maximal dose tested is chosen to be significantly higher than the maximum tolerated doses, and hence we do not expect this saturation to affect the accuracy of our model at clinically relevant doses.

3.2 Evolutionary stability measured by maximum eigenvalues

Figures (S8) and (3) (main text) depict maximum eigenvalue decompositions of HGF- and HGF+ tumors and describe the set of initial NSCLC populations, if present can lead to tumor progression upon initiation of constant (non-switching) combination treatments. For the evolutionary dynamics:

$$\dot{x} = (A - D_\ell) x \tag{S15}$$

where $x \in \mathbb{R}^n$ is a vector of concentrations of n NSCLC subpopulations, $\dot{x} \in \mathbb{R}^n$ is their rate of change over time, $A \in \mathbb{R}^{n \times n}$ is a matrix that represents the growth and mutation dynamics and $D_\ell \in \mathbb{R}^{n \times n}$ is a diagonal matrix that represents the corresponding drug effect of one constant drug treatment on the rate of change of NSCLC cells. If all eigenvalues are negative then Equation (S15) is said to be *stable*. In the case of NSCLC evolutionary dynamics corresponding to Equation (1), *stability* refers to tumor reduction, and *instability* refers to tumor progression. In section 3.1, we made the assumption that mutation rates are one directional, hence the A matrix in Equation (S15) is lower triangular and the eigenvalues of $A - D_\ell$ are exactly equal to its diagonal entries. For each NSCLC subpopulation, we take the maximum eigenvalue for each evolutionary branch downstream of the population and define this as *evolutionary stability.* This maximum eigenvalue represents the worst case stability if the particular population is present upon treatment initiation - a positive maximum eigenvalue indicates that the presence of the cell subpopulation in the tumor upon initiation of treatment is likely to cause therapeutic failure. A negative maximum eigenvalue indicates that the presence of the particular subpopulation will not outgrow or evolve in the presence of therapy.

3.3 Robustness analysis

Sensitivity to drug perturbations. To analyze the effect of dose reductions on the robustness of constant and switching treatment strategies, we perturbed the drug concentrations and calculated the ratio of final cost and initial cost (Figures (S9)) . We rewrite Equation (S1) for one cell x_i and one drug ℓ_j to illustrate how a drug perturbation $\delta \in \mathbb{R}^{[0,1]}$ is modeled:

$$\dot{x}_i = r_i q_{ii} x_i + \sum_{k \neq i}^{n} r_i q_{ik} x_k - \gamma_{j,i} \frac{(\delta \ell)^{n_{j,i}}}{(\delta \ell)^{n_{j,i}} + K_{j,i}^{n_{j,i}}} x_i \tag{S16}$$

The fold change FC_f in total population from day 0 to day N for a sequence of drugs $\{\ell(k)\}_{k=1}^{N/\tau-1}$ defining a switching strategy over a time horizon N, and initial tumor population $x_0 = x(0)$ is calculated by

$$FC_f = \frac{C_f}{\sum x_0} = \frac{e^{A(\{\ell(k)\}_{k=1}^{N/\tau-1})} x_0}{\sum x_0}. \tag{S17}$$

If $FC_f < 1$, the treatment strategy $\{\ell(k)\}_{k=1}^{N/\tau-1}$ is effective for NSCLC populations for the duration of the time horizon N, $FC_f > 1$ indicates progression.

3.4 Implementation

The evolutionary dynamics model and simulations were implemented using python, scipy and numpy (versions 3.5.1, 0.17.0, 1.9.3) and pandas version 0.17.0 was used for data parsing. Data fitting for experimentally derived cell growth and drug dose response data was performed with Matlab version 8.3.0.532 using the non linear least squares method.

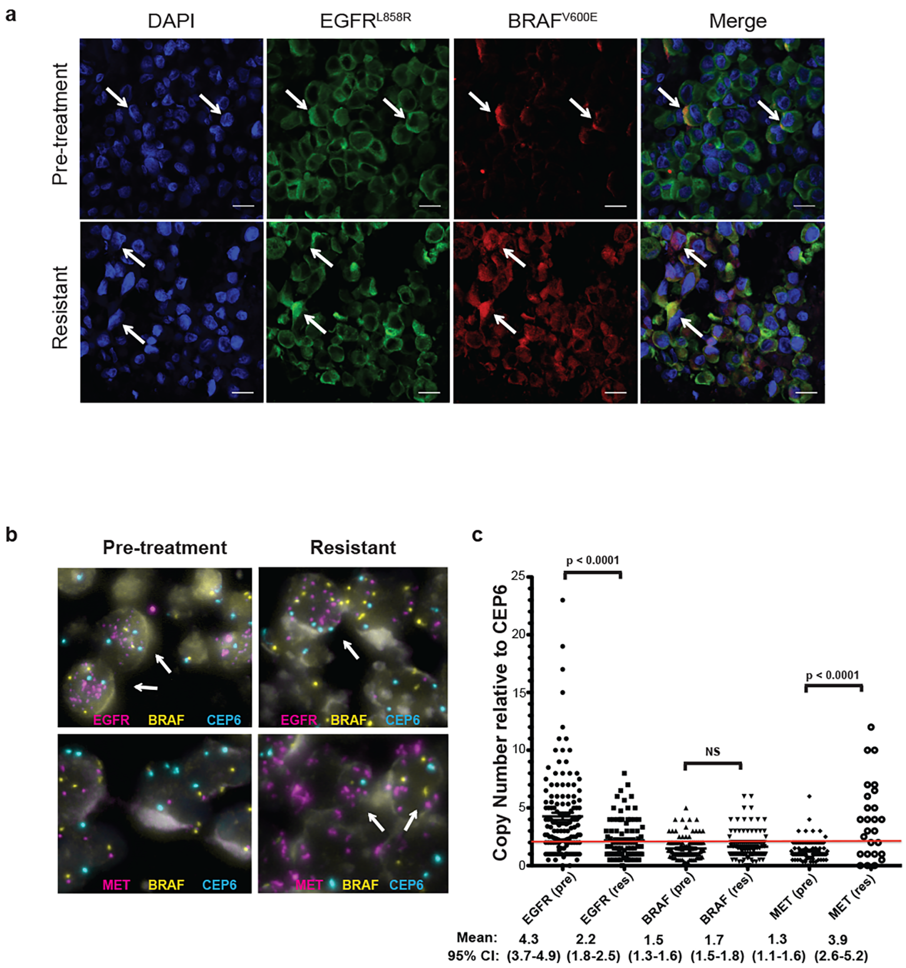

Figure S1: Immunofluorescence and FISH analysis of pre-treatment and erlotinib-resistant tumors. (a) Immunofluorescence of pre-treatment and erlotinib resistant tumors demonstrating expression of $EGFR^{L858R}$ (green) and $BRAF^{V600E}$ (red) in tumor cells prior to therapy and in the resistant tumor. Examples of cells expressing both $EGFR^{L858R}$ and $BRAF^{V600E}$ cell in the pre-treatment and resistant tumor samples are indicated by the white arrow. Scale bar = 10 microns. (b) Fluorescence in situ hybridization (FISH) performed on pre-treatment (left) and erlotinib resistant (right) tumor samples using probes for EGFR (red, upper), BRAF (gold), and CEP6 (aqua), and MET (red, lower). Representative cells with EGFR or MET amplification are indicated by white arrows in the upper and lower panels, respectively. (c) ratio of EGFR, BRAF and MET to CEP6 identifies focal amplification of EGFR in the pre-treatment tumor and focal amplification of MET in the resistant tumor. Red line denotes a copy number:CEP6 ratio of 2.0, indicative of amplification. P-value was determined by two-tailed, unpaired T-test comparing pre-treatment and resistant tumors.

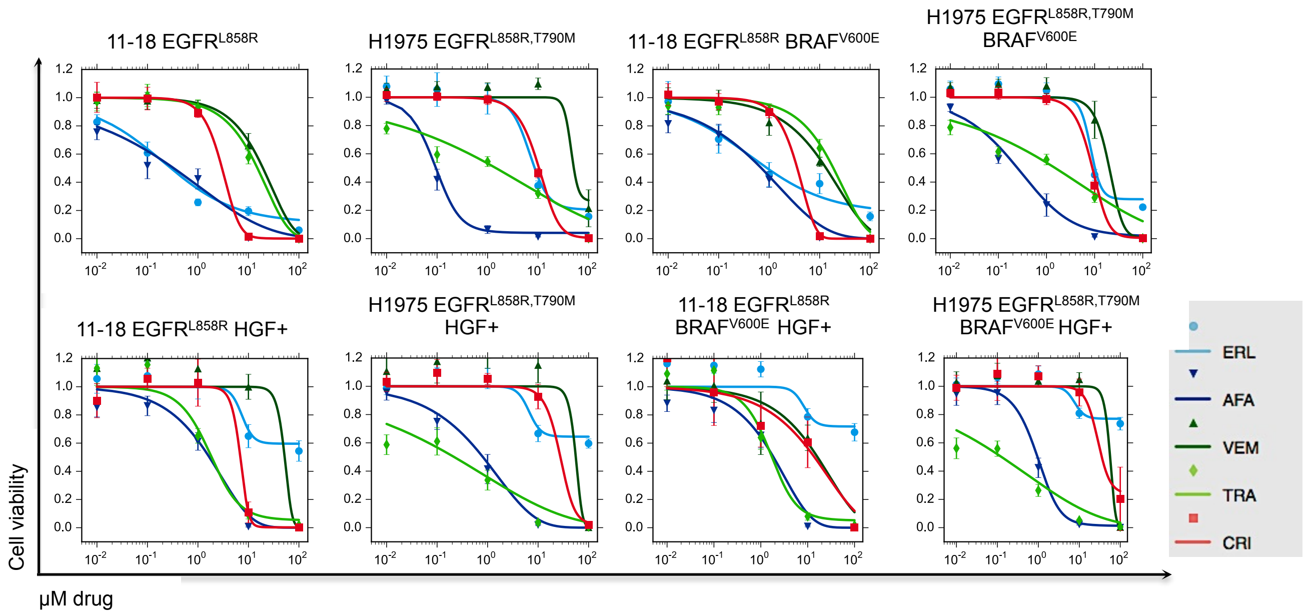

Figure S2: Experimentally derived erlotinib, afatinib, vemurafenib, trametinib and crizotinib dose response curves for 11-18 EGFR^{L858R}, 11-18 EGFR^{L858R} BRAF^{V600E}, H1975 EGFR^{L858R,T790M} H1975 EGFR^{L858R,T790M} BRAF^{V600E} cell lines, and either 0 or 50 ng/ml human growth factor (HGF) and fit with $\gamma\frac{[\ell]^n}{[\ell]^n+K^n}$ where γ is the maximum inhibition, $[\ell]$ is the EGFR TKI concentration, n is the Hill coefficient and K is the half maximal inhibitory concentration (IC50).

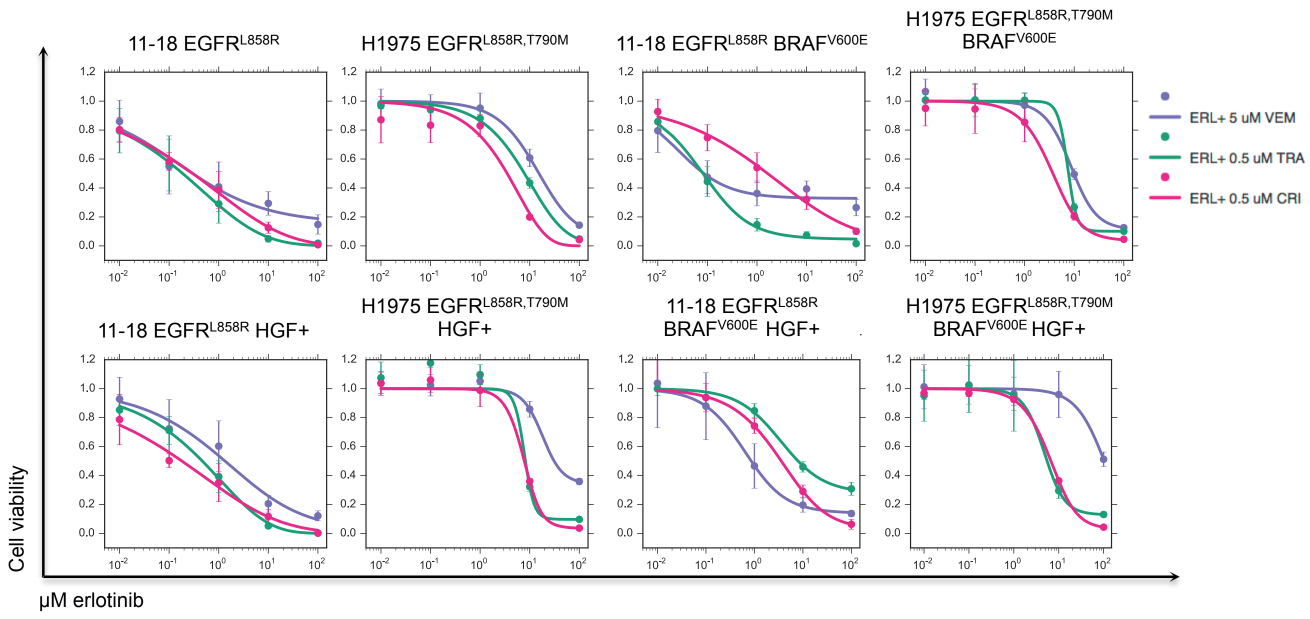

Figure S3: Experimentally derived dose response curves for erlotinib in combination with 5 μM vemurafenib, 0.5 μM trametinib and 0.5 μM crizotinib for 11-18 EGFR^{L858R}, 11-18 EGFR^{L858R}BRAF^{V600E}, H1975 EGFR^{L858R,T790M} H1975 EGFR^{L858R,T790M} BRAF^{V600E} cell lines, and either 0 or 50 ng/ml human growth factor (HGF) and fit with $\gamma\frac{[\ell]^n}{[\ell]^n+K^n}$ where γ is the maximum inhibition, $[\ell]$ is the EGFR TKI concentration, n is the Hill coefficient and K is the half maximal inhibitory concentration (IC50).

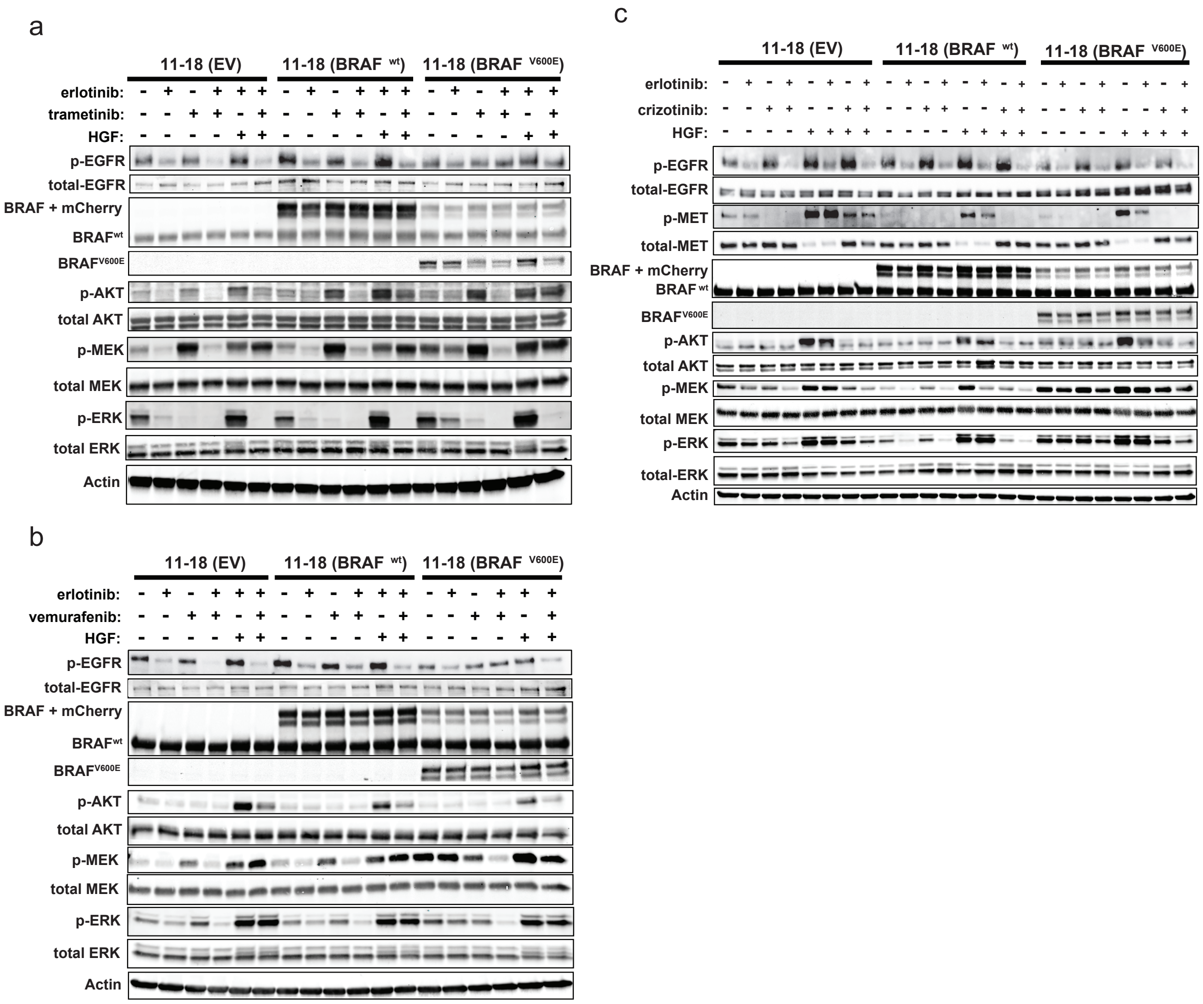

Figure S4: Western blot analysis of cell lysates obtained from 11-18 cell line, treated with drugs and/or HGF as indicated, and probed for the indicated proteins. Corresponding full length gels are shown in S13, S14 and S15.

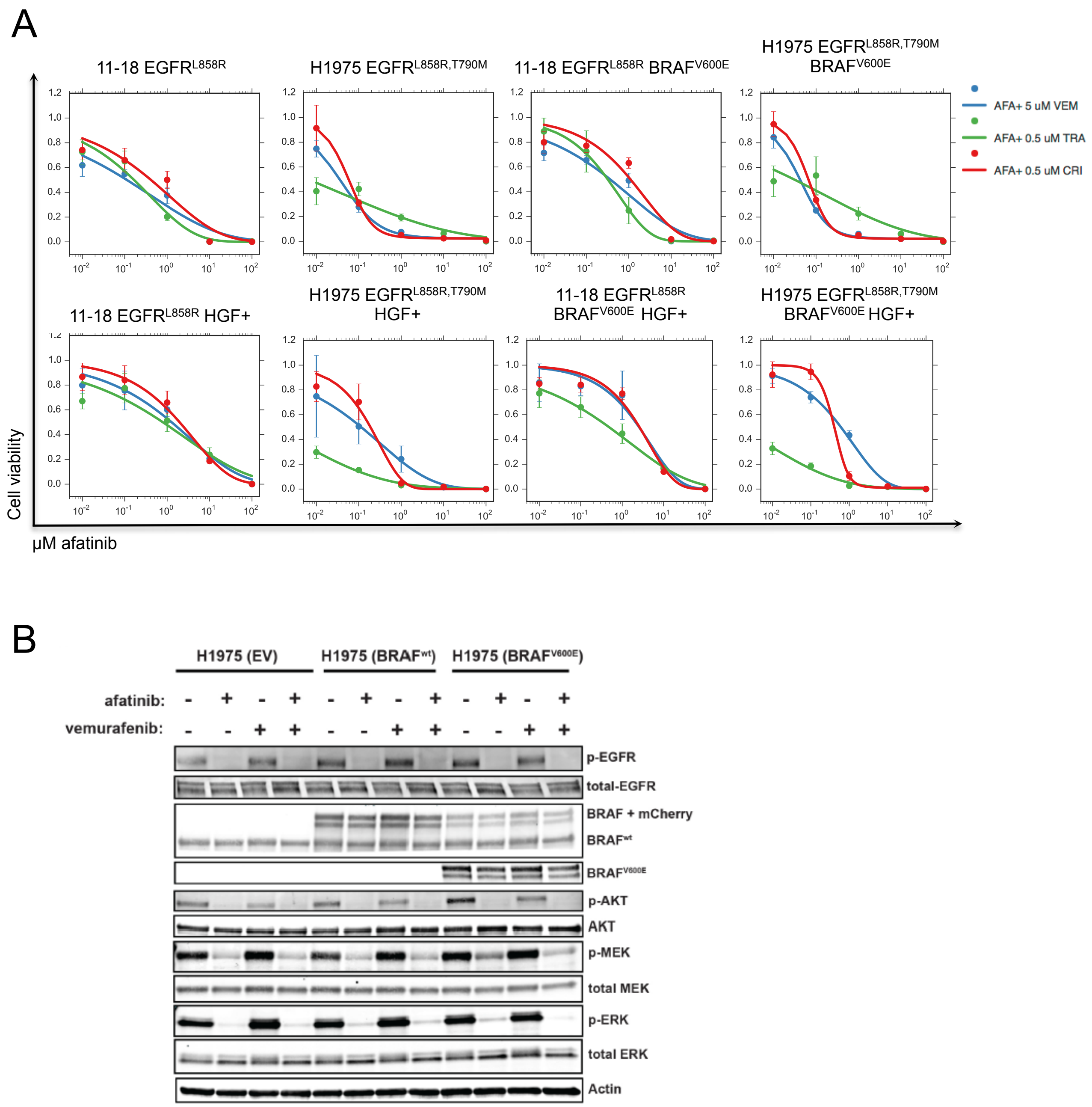

Figure S5: A) Experimentally derived dose response curves for afatinib in combination with 5 μM vemurafenib, 0.5 μM trametinib and 0.5 μM crizotinib for 11-18 EGFR^{L858R}, 11-18 EGFR^{L858R} BRAF^{V600E}, H1975 EGFR^{L858R,T790M} H1975 EGFR^{L858R,T790M} BRAF^{V600E} cell lines, and either 0 or 50 ng/ml human growth factor (HGF) and fit with $\gamma\frac{[\ell]^n}{[\ell]^n+K^n}$ where γ is the maximum inhibition, $[\ell]$ is the EGFR TKI concentration, n is the Hill coefficient and K is the half maximal inhibitory concentration (IC50). (B) Western blot analysis of cell lysates obtained from H1975 cell lines, treated with drugs and/or HGF as indicated, and probed for the indicated proteins. Corresponding full length gel is shown in S16.

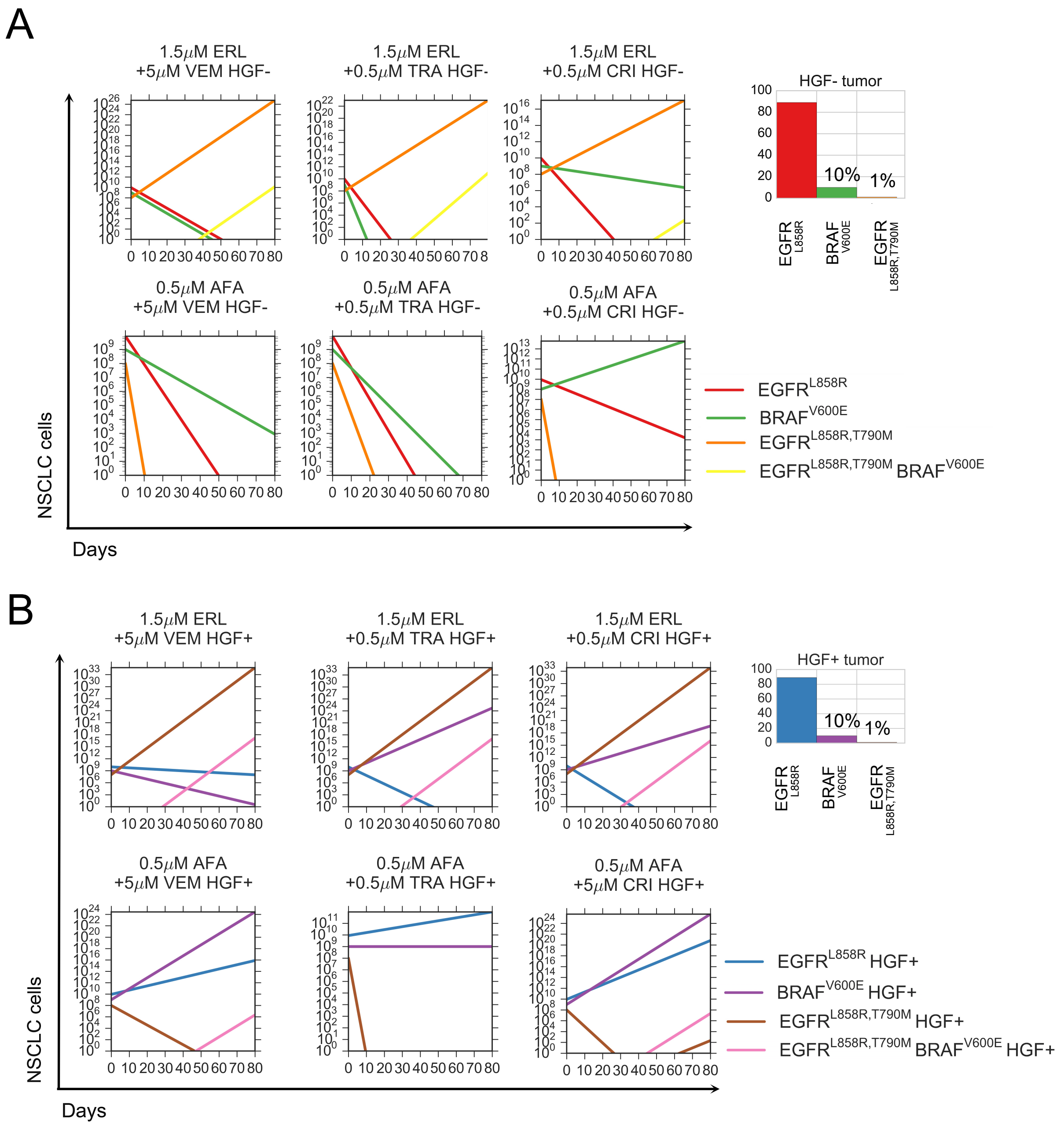

Figure S6: Simulations of the NSCLC model for constant combinations of 0.5 μM afatinib or 1.5 μM erlotinib with either 0.5 μM trametinib, 0.5 μM crizotinib or 5 μM vemurafenib for a tumor comprised of 89% 11-18 $EGFR^{L858R}$, 10% 11-18 $EGFR^{L858R}$, $BRAF^{V600E}$ and 1% H1975 $EGFR^{L858R\ T790M}$, and treated with HGF (B) or without HGF (A).

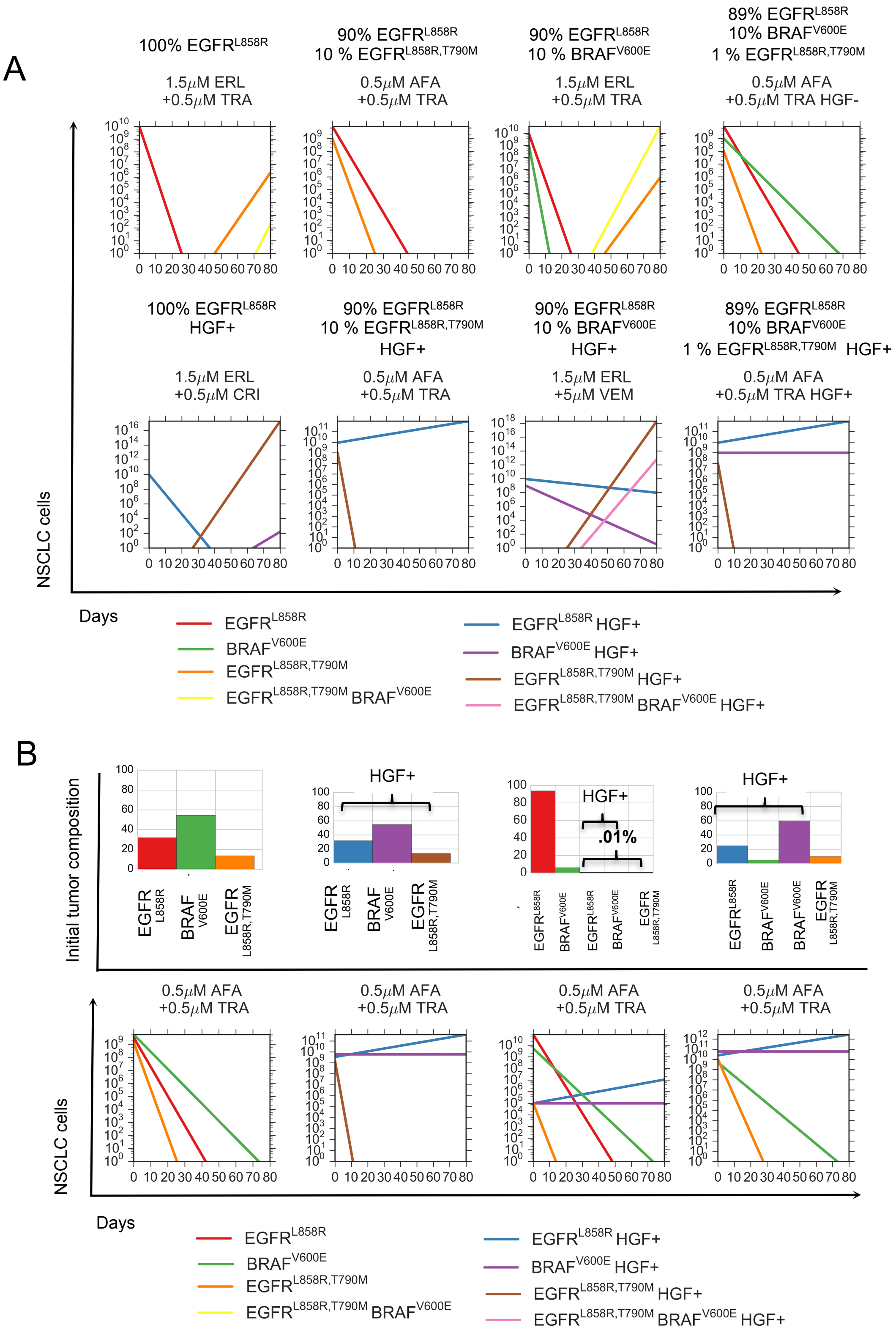

Figure S7: Simulations of the NSCLC model for the optimal 30 day constant combinations found by Algorithm (4) with 0.5 μM afatinib or 1.5 μM erlotinib with either 0.5 μM trametinib, 0.5 μM crizotinib or 5 μM vemurafenib for the relatively low (A) initial tumor heterogeneity or with (B) high initial tumor heterogeneity.

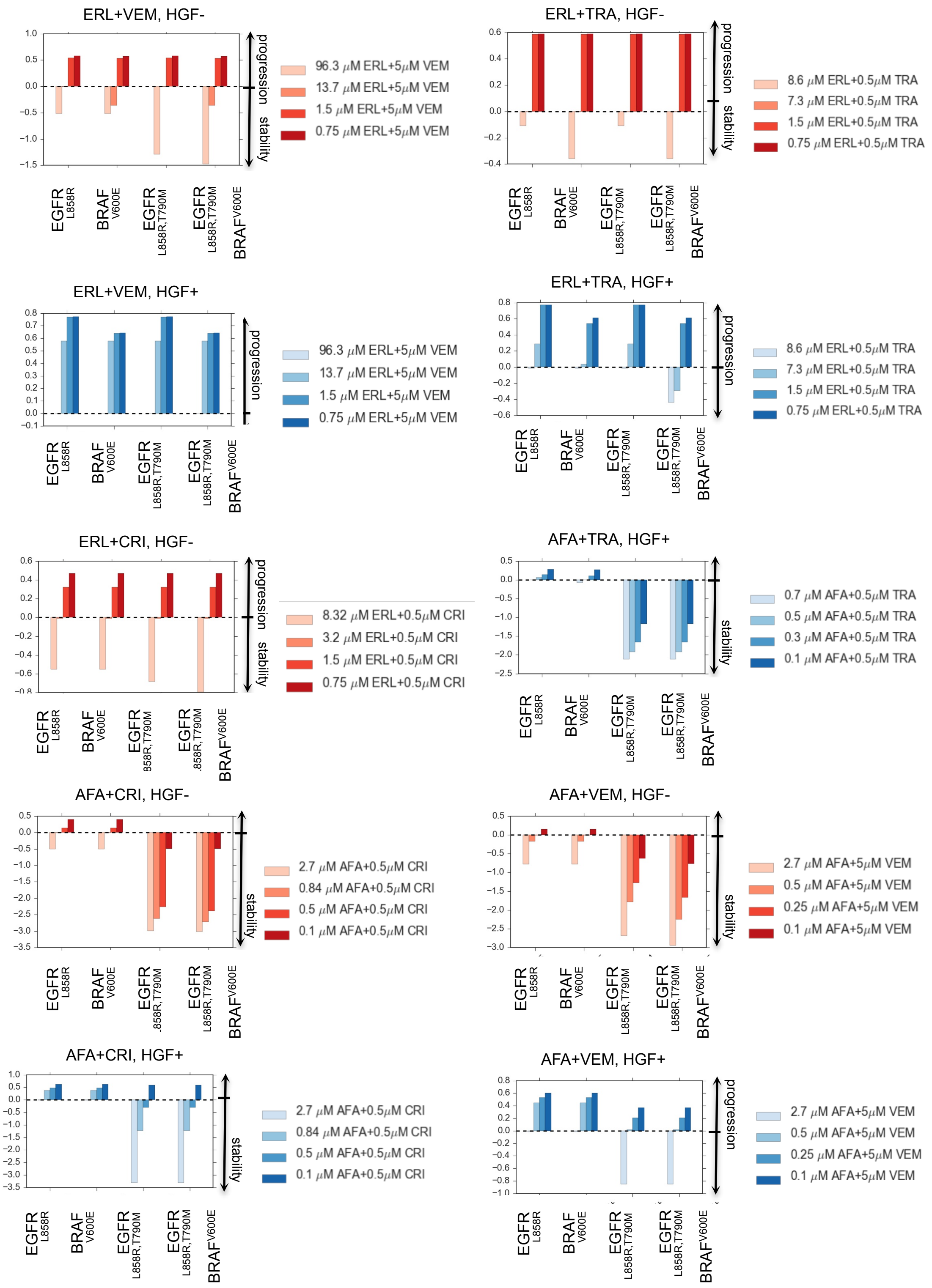

Figure S8: Classification of initial tumor compositions via eigenvalue decompositions describe the initial tumor populations that can destabilize of the evolutionary dynamics in the presence of either erlotinib or afatinib and either 0.5 μM trametinib, 0.5 μM crizotinib or 5 μM vemurafenib.

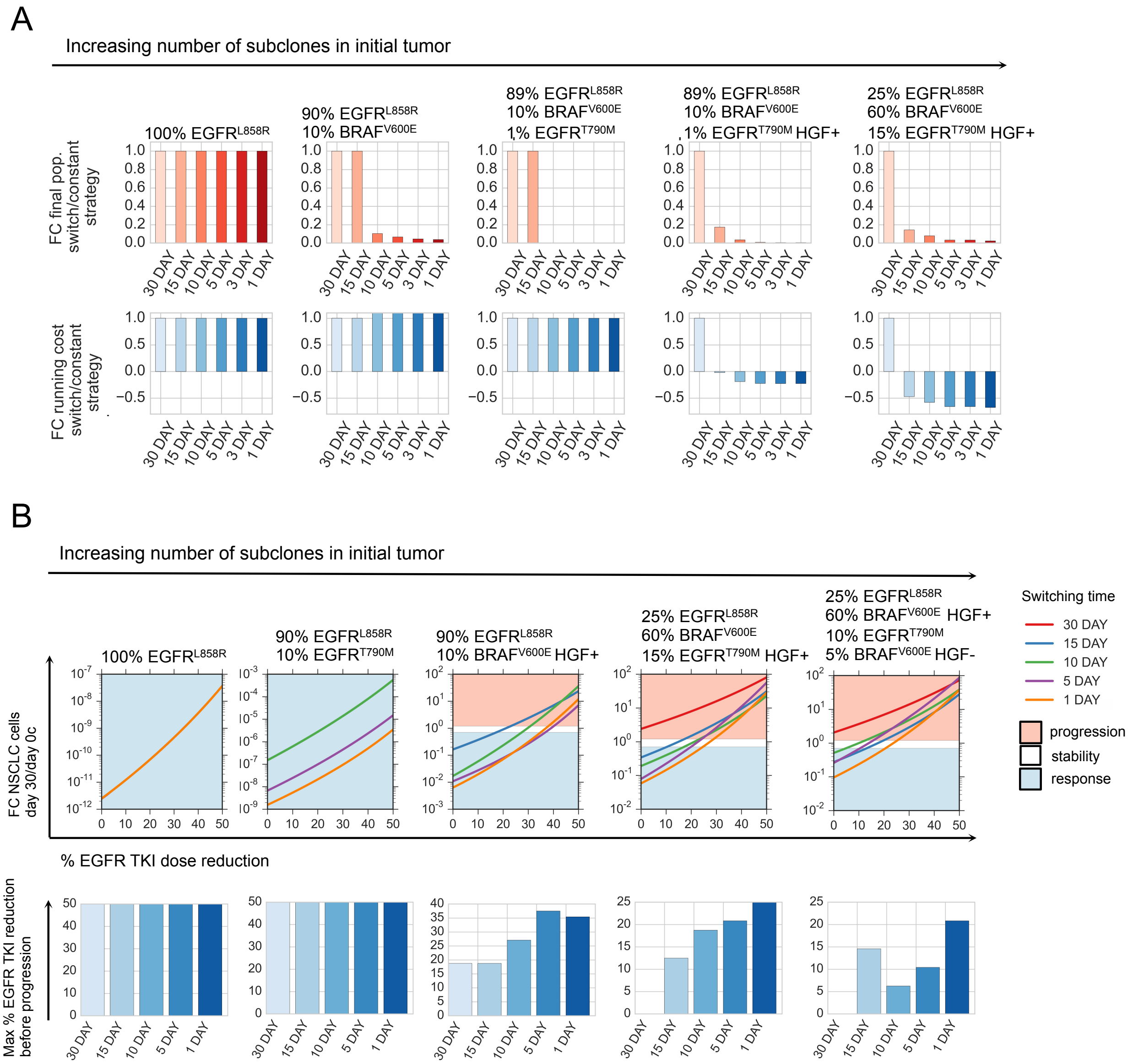

Figure S9: A) Fold change in NSCLC population at day 30 versus day 0, over the course of the optimal 30, 15, 10, 5, 3, and 1 day treatment strategies solved by algorithm 1 (SI), for indicated tumor compositions, normalized by fold change in NSCLC population for the constant 30 day treatment strategy (Red). (Blue) Sum of fold change in the average cost for indicated tumor compositions and corresponding optimal 30, 15, 10, 5, 3, and 1 day treatment strategies. B) (Above) Fold change in number of NSCLC cells between day 0 and day 30, as a function of percent EGFR TKI dose reduction for the optimal 30, 15, 10, 5 and 1 day strategies solved by algorithm 1 (SI) for indicated tumor compositions. Shaded blue areas indicate the region of the perturbation space where the treatment strategy reduces the size of the initial tumor (stable). The shaded red area indicates the region of the perturbation space where the treatment strategy increases the size of the original tumor at day 30 (unstable). (Below) The maximum percent EGFR TKI dose reduction sustainable before the treatment is no longer effective (the tumor progresses).

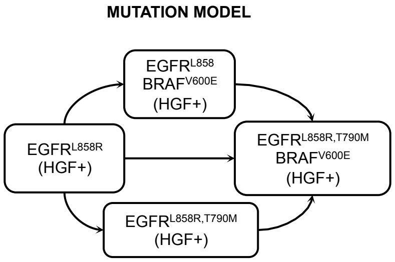

Figure S10: The EGFR-mutant lung adenocarcinoma mutation model used in this study.

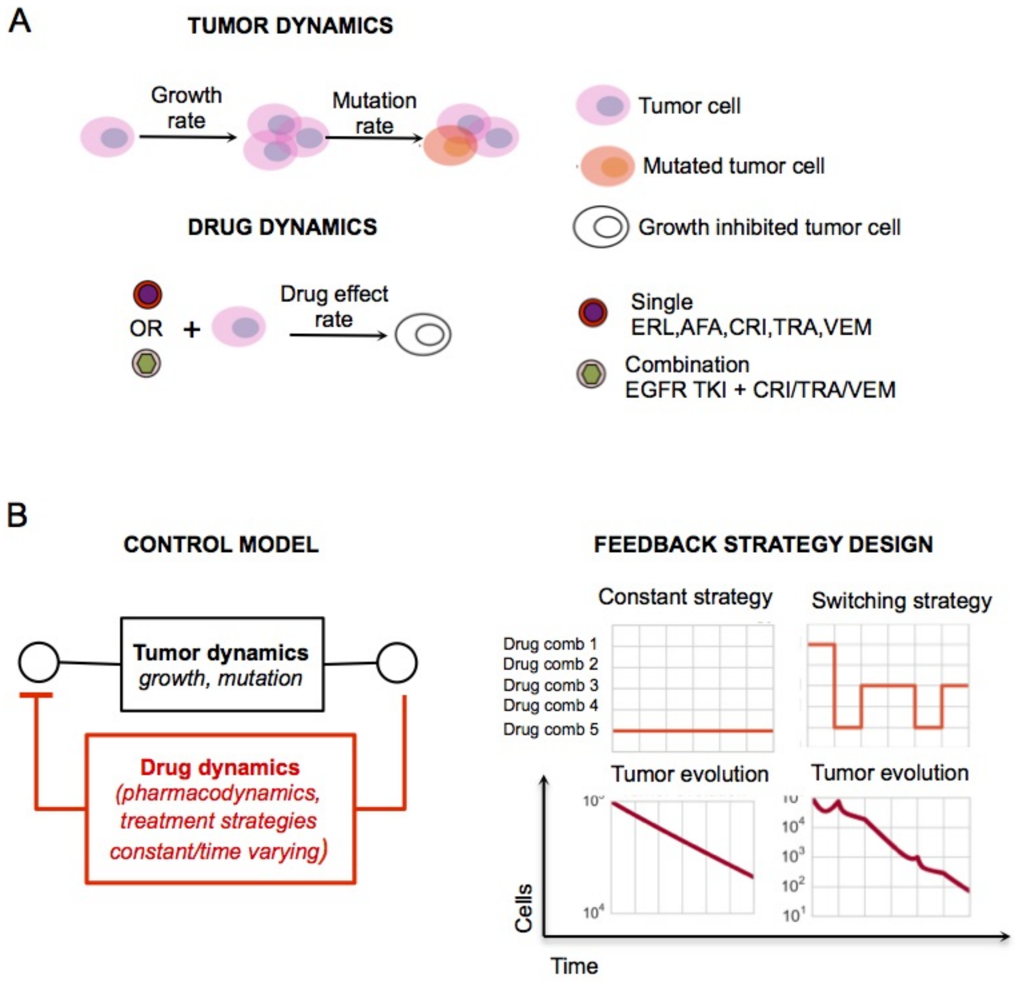

Figure S11: Designing treatment strategies to control tumor cell dynamics. (A) A depiction of the growth, mutation and drug effect model representing the evolutionary dynamics of lung adenocarcinoma in the presence of small molecule inhibitors, erlotinib (ERL), afatinib (AFA), crizotinib (CRI), trametinib (TRA) and vemurafenib (VEM). The corresponding ordinary differential equation model (ODE) is specified in mathematical detail in the Supplementary Information, Equation (S1). Drug effect curves were determined for 11-18 and H1975 cell lines specified for both single drugs and combinations of varying concentrations of one EGFR TKI (erlotinib or afatinib), with fixed concentrations of either 5 μM vemurafenib, 0.5 μM trametinib or 0.5 μM crizotinib (SI, Fig. S2-S5). (B) The design of constant or switching feedback strategies to control the dynamics of lung adenocarcinoma is approached as an optimal control problem. The treatment strategy design algorithm (SI, Section 2) solves for feedback strategies that minimize tumor cell growth over the course of the treatment..

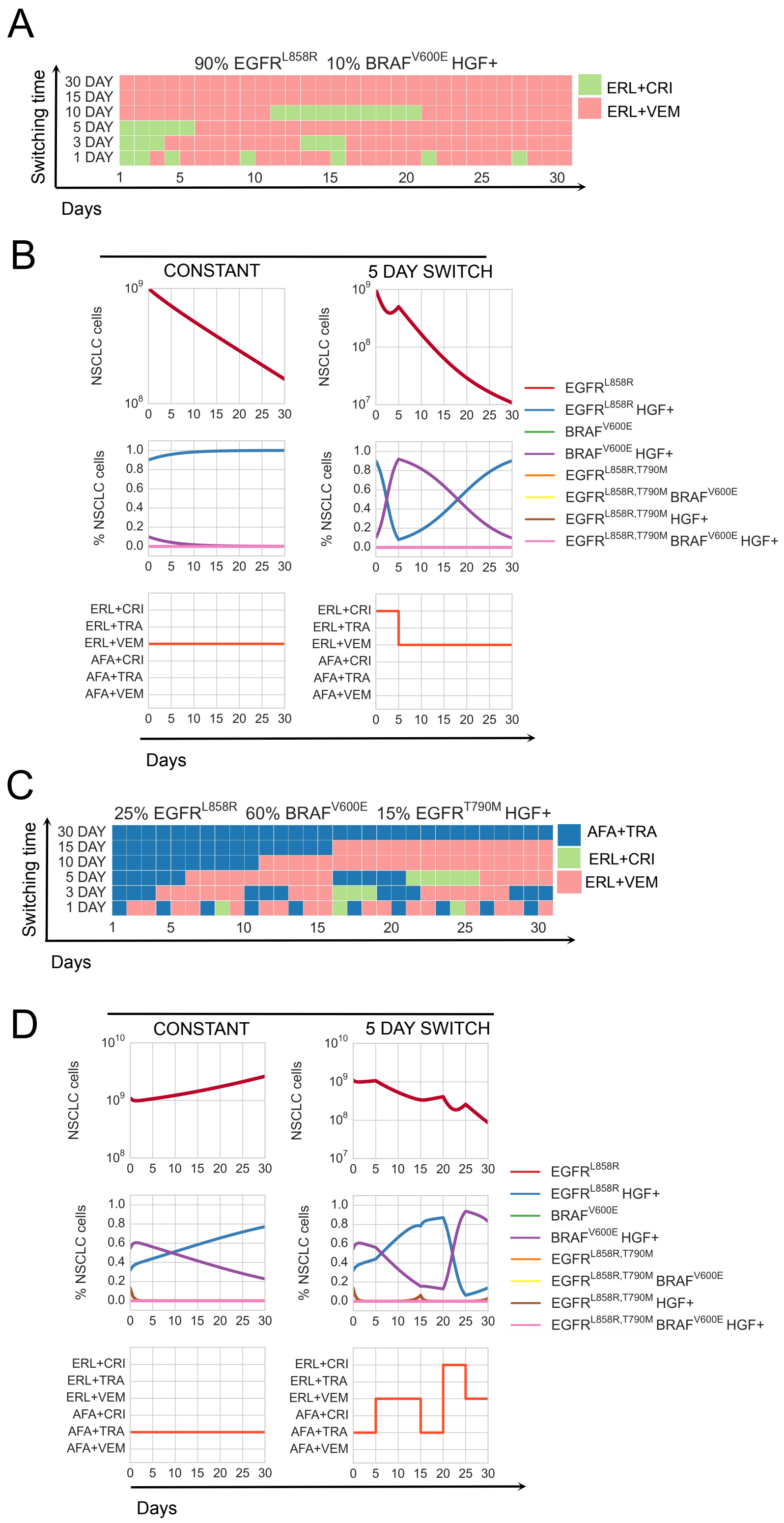

Figure S12: Optimal drug scheduling strategies solved by Algorithm 1 (SI, Section 2.2) for representative initial tumor cell distributions (A),(C), for a 30 day timeframe and 30, 15, 10, 5, 3 and 1 day minimum switching horizons, give one EGFR TKI, either 1.5 μM erlotinib (ERL) or 0.5 μM afatinib (AFA) in combination with either 5 μM vemurafenib (VEM), 0.5 μM trametinib (TRA) or 0.5 μM crizotinib (CRI) and corresponding simulations (B),(D) of the lung adenocarcinoma evolutionary dynamics for a subset of optimal drug scheduling strategies.

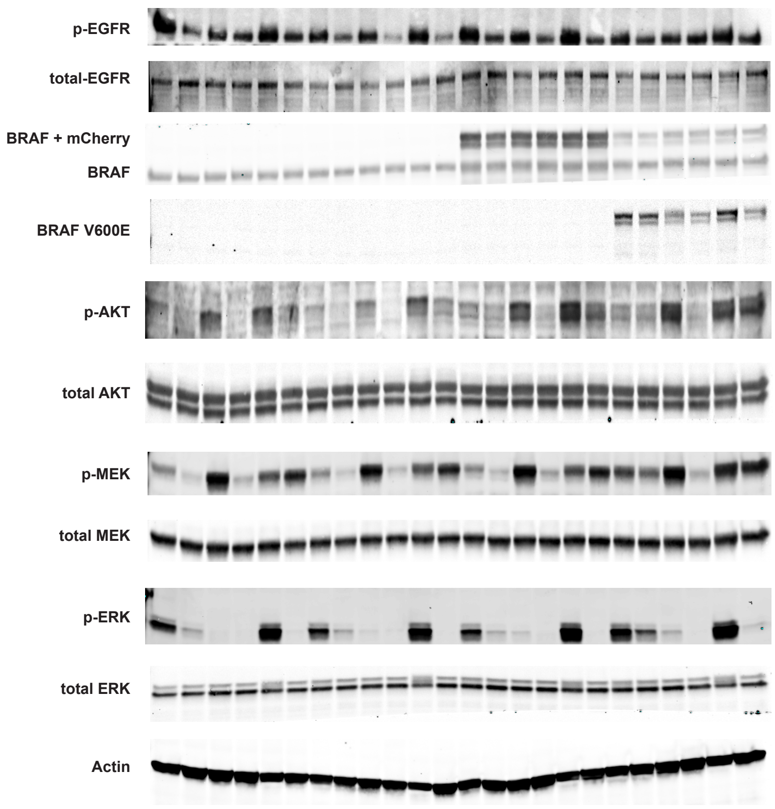

Figure S13: Uncropped western blots for Figure S4A (note, blots were cut prior to incubation with primary antibody).

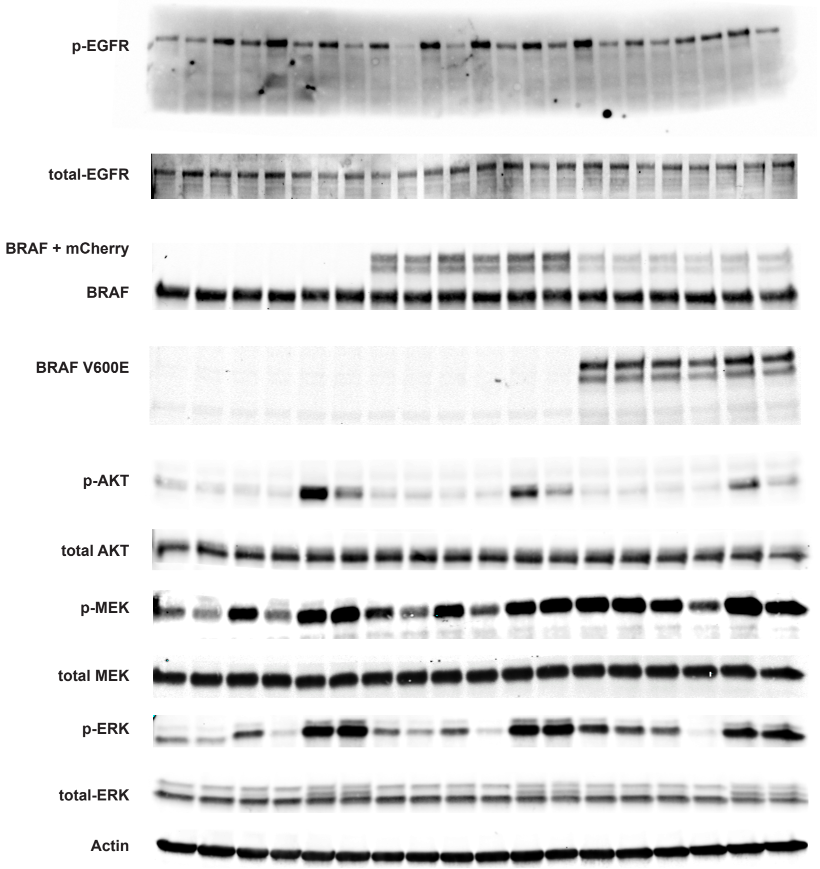

Figure S14: Uncropped western blots for Figure S4B (note, blots were cut prior to incubation with primary antibody).

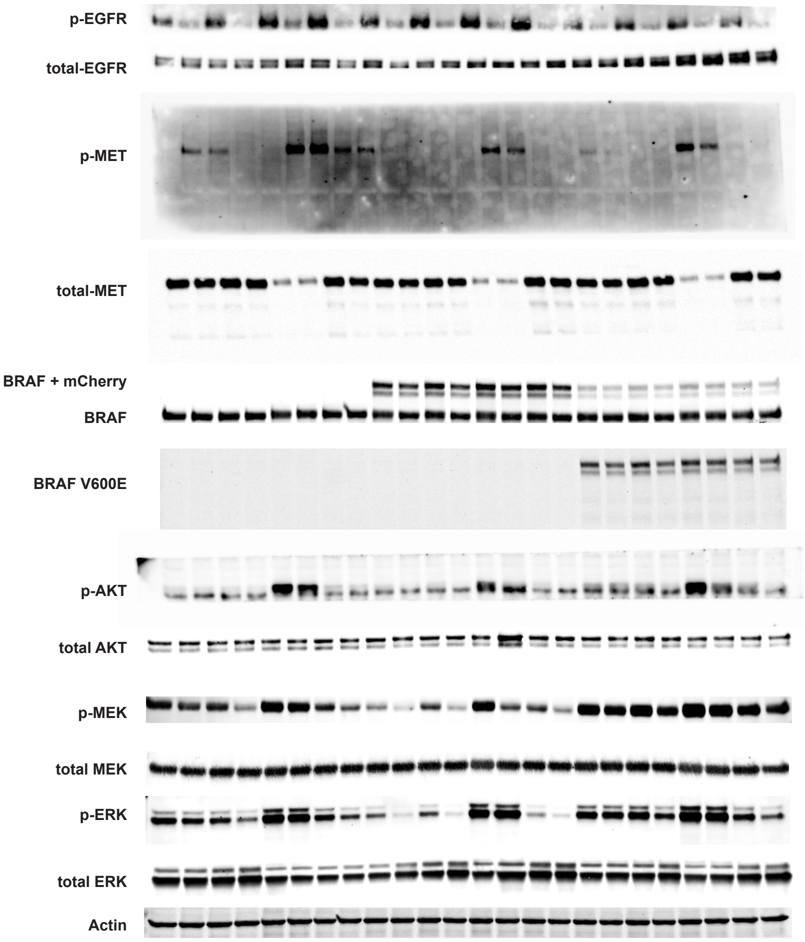

Figure S15: Uncropped western blots for Figure S4C (note, blots were cut prior to incubation with primary antibody).

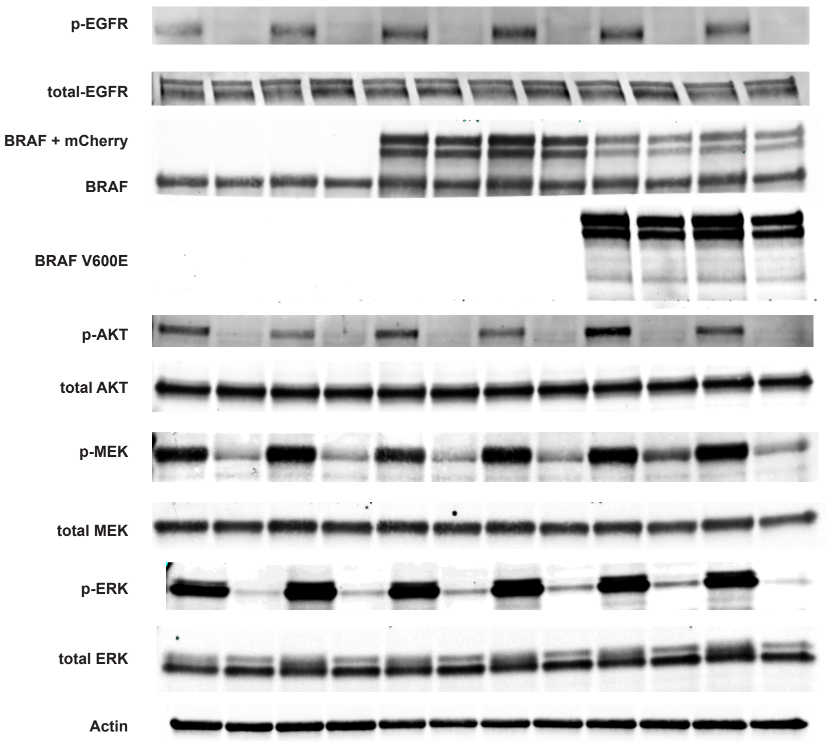

Figure S16: Uncropped western blots for Figure S5B (note, blots were cut prior to incubation with primary antibody).

Cell name	Growth rate, day^{-1}
11-18 $EGFR^{L858R}$	0.58
11-18 $EGFR^{L858R}$ HGF+	0.67
11-18 $EGFR^{L858R}$ $BRAF^{V600E}$	0.60
11-18 $EGFR^{L858R}$ $BRAF^{V600E}$ HGF+	0.70
H1975 $EGFR^{L858R,T790M}$	0.63
H1975 $EGFR^{L858R,T790M}$ $BRAF^{V600E}$	0.59
H1975 $EGFR^{L858R,T790M}$ HGF+	0.77
H1975 $EGFR^{L858R,T790M}$ $BRAF^{V600E}$ HGF+	0.64

Table 1: Experimentally derived growth rates in parental and engineered 11-18 $EGFR^{L858R}$-positive lung adenocarcinoma cells and treated with or without HGF, fit with Equation (S8).

	IC50 in μM				
Cellname	Erlotinib	Afatinib	Crizotinib	Trametinib	Vemurafenib
11-18 $EGFR^{L858R}$	0.19	0.20	2.72	12.69	16.38
11-18 $EGFR^{L858R}$ HGF+	7.93	1.33	6.81	1.59	50.18
11-18 $EGFR^{L858R}$ $BRAF^{V600E}$	0.91	0.49	3.25	15.59	10.60
11-18 $EGFR^{L858R}$ $BRAF^{V600E}$ HGF+	8.74	1.49	10.54	1.49	12.64
H1975 $EGFR^{L858R,T790M}$	7.54	0.08	9.33	0.76	48.31
H1975 $EGFR^{L858R,T790M}$ $BRAF^{V600E}$	9.32	0.18	8.18	0.82	18.64
H1975 $EGFR^{L858R,T790M}$ HGF+	7.04	0.60	25.59	0.12	53.89
H1975 $EGFR^{L858R,T790M}$ $BRAF^{V600E}$ HGF+	7.97	0.82	31.83	0.06	54.48

Table 2: Drug sensitivity as measured by the IC50 of erlotinib, afatinib, vemurafenib, trametinib and crizotinib in parental and engineered 11-18 $EGFR^{L858R}$-positive lung adenocarcinoma cells.

	IC50 Erlotinib in μM		
Cell name	+0.5 μM Crizotinib	+0.5 μM Trametinib	+5 μM Vemurafenib
11-18 $EGFR^{L858R}$	0.30	0.19	0.30
11-18 $EGFR^{L858R}$ HGF+	0.18	0.47	1.34
11-18 $EGFR^{L858R}$ $BRAF^{V600E}$	1.64	0.08	0.09
11-18 $EGFR^{L858R}$ $BRAF^{V600E}$ HGF+	3.58	7.95	0.86
H1975 $EGFR^{L858R,T790M}$	3.51	7.83	15.39
H1975 $EGFR^{L858R,T790M}$ $BRAF^{V600E}$	3.71	7.68	9.86
H1975 $EGFR^{L858R,T790M}$ HGF+	7.78	8.20	31.01
H1975 $EGFR^{L858R,T790M}$ $BRAF^{V600E}$ HGF+	6.70	5.50	103.67

Table 3: Drug sensitivity as measured by the IC50 of erlotinib in combination with 5 μM vemurafenib, 0.5 μM trametinib and 0.5 μM crizotinib in parental and engineered 11-18 $EGFR^{L858R}$-positive lung adenocarcinoma cells.

	IC50 Afatinib in μM		
Cell name	+0.5 μM Crizotinib	+0.5 μM Trametinib	+5 μM Vemurafenib
11-18 $EGFR^{L858R}$	0.42	0.17	0.11
11-18 $EGFR^{L858R}$ HGF+	1.96	0.79	1.27
11-18 $EGFR^{L858R}$ $BRAF^{V600E}$	1.10	0.32	0.37
11-18 $EGFR^{L858R}$ $BRAF^{V600E}$ HGF+	2.60	0.48	2.54
H1975 $EGFR^{L858R,T790M}$	0.06	0.01	0.04
H1975 $EGFR^{L858R,T790M}$ $BRAF^{V600E}$	0.07	0.03	0.04
H1975 $EGFR^{L858R,T790M}$ HGF+	0.19	0.00	0.11
H1975 $EGFR^{L858R,T790M}$ $BRAF^{V600E}$ HGF+	0.41	0.00	0.61

Table 4: Drug sensitivity as measured by the IC50 of afatinib in combination with 5 μM vemurafenib, 0.5 μM trametinib and 0.5 μM crizotinib in parental and engineered 11-18 $EGFR^{L858R}$-positive lung adenocarcinoma cells.

	Erlotinib			Afatinib		
Cell name	γ	n	K	γ	n	K
11-18 EGFR^{L858R}	2.59	0.54	1.22	73.45	0.32	424540.00
11-18 EGFR^{L858R} HGF+	0.61	3.81	7.93	1885.80	0.70	106610.00
11-18 EGFR^{L858R} BRAF^{V600E}	1.72	0.53	1.90	297.20	0.46	269500.00
11-18 EGFR^{L858R} BRAF^{V600E} HGF+	0.39	3.48	8.74	590.76	0.72	18112.00
H1975 EGFR^{L858R,T790M}	1.87	2.23	9.54	3.77	1.49	0.22
H1975 EGFR^{L858R,T790M} BRAF^{V600E}	1.50	3.86	9.70	5.14	0.62	3.67
H1975 EGFR^{L858R,T790M} HGF+	0.52	3.67	7.04	250.96	0.56	21189.00
H1975 EGFR^{L858R,T790M} BRAF^{V600E} HGF+	0.31	3.50	7.97	5.14	1.26	3.57

Table 5: Differential equation parameters derived using Equation (S12), corresponding to experimentally derived dose response curves of erlotinib and afatinib for parental and engineered 11-18 EGFR^{L858R}-positive lung adenocarcinoma cells.

	Crizotinib			Trametinib			Vemurafenib		
Cell name	γ	n	K	gamma	n	K	γ	n	K
11-18 EGFR^{L858R}	8.80	1.84	10.36	207.05	0.94	5504.20	825.97	0.95	27377.00
11-18 EGFR^{L858R} HGF+	6.14	3.89	11.57	3.47	1.29	4.67	9.81	4.42	89.86
11-18 EGFR^{L858R} BRAF^{V600E}	74.60	1.59	61.34	1135.50	0.88	67225.00	672.41	0.69	236280.00
11-18 EGFR^{L858R} BRAF^{V600E} HGF+	235.65	0.57	270080.00	3.51	1.36	4.17	229.34	0.64	104240.00
H1975 EGFR^{L858R,T790M}	6.98	1.68	34.62	28.40	0.26	1064100.00	1.56	5.88	50.20
H1975 EGFR^{L858R,T790M} BRAF^{V600E}	6.52	1.99	23.88	35.42	0.28	1223700.00	6.92	2.35	47.51
H1975 EGFR^{L858R,T790M} HGF+	4.66	2.51	51.31	47.17	0.27	900880.00	9.35	4.95	89.74
H1975 EGFR^{L858R,T790M} BRAF^{V600E} HGF+	1.69	2.79	36.20	38.13	0.25	595280.00	8.62	4.92	89.37

Table 6: Differential equation parameters derived using Equation (S12), corresponding to experimentally derived dose response curves of crizotinib, trametinib and vemurafenib for parental and engineered 11-18 EGFR^{L858R}-positive lung adenocarcinoma cells.

	Erlotinib+0.5 μM Crizotinib			Erlotinib+0.5 μM Trametinib			Erlotinib+5 μM Vemurafenib		
Cell name	γ	n	K	γ	n	K	γ	n	K
11-18 EGFR^{L858R}	49.60	0.32	215970.00	23.91	0.38	2057.10	1.87	0.45	0.96
11-18 EGFR^{L858R} HGF+	13.87	0.31	2167.30	197.39	0.44	180410.00	4.03	0.44	46.41
11-18 EGFR^{L858R} BRAF^{V600E}	5.02	0.38	204.43	3.10	0.77	0.41	1.11	0.85	0.05
11-18 EGFR^{L858R} BRAF^{V600E} HGF+	3.79	0.75	26.45	1.28	0.95	6.69	1.97	0.95	1.63
H1975 EGFR^{L858R,T790M}	990.17	0.74	65062.00	6.43	0.80	110.13	3.01	0.97	53.62
H1975 EGFR^{L858R,T790M} BRAF^{V600E}	3.40	1.22	11.30	2.29	4.29	9.33	2.20	1.54	16.34
H1975 EGFR^{L858R,T790M} HGF+	3.37	2.08	14.90	2.34	4.05	10.15	1.07	2.12	23.25
H1975 EGFR^{L858R,T790M} BRAF^{V600E} HGF+	3.65	1.22	21.97	2.06	1.76	8.08	3.04	1.27	271.56

Table 7: Differential equation parameters as derived using Equation (S12), corresponding to experimentally derived dose response curves of erlotinib in combination with either 0.5 μM crizotinib, 0.5 μM trametinib or 5 μM vemurafenib for parental and engineered 11-18 EGFR^{L858R}-positive lung adenocarcinoma cells.

	Afatinib+0.5 μM Crizotinib			Afatinib+0.5 μM Trametinib			Afatinib+5 μM Vemurafenib		
**Cell name**	**γ**	**n**	**K**	**γ**	**n**	**K**	**γ**	**n**	**K**
11-18 $EGFR^{L858R}$	102.02	0.36	363520.00	190.37	0.42	116910.00	41.09	0.27	314410.00
11-18 $EGFR^{L858R}$ HGF+	212.01	0.49	209360.00	42.67	0.29	977910.00	66.90	0.36	344940.00
11-18 $EGFR^{L858R}$ $BRAF^{V600E}$	310.67	0.52	132530.00	141.93	0.60	2315.40	101.84	0.35	673450.00
11-18 $EGFR^{L858R}$ $BRAF^{V600E}$ HGF+	1440.80	0.69	161180.00	54.46	0.31	621930.00	311.18	0.61	57467.00
H1975 $EGFR^{L858R,T790M}$	3.75	1.25	0.19	19.69	0.18	572790.00	3.87	0.77	0.26
H1975 $EGFR^{L858R,T790M}$ $BRAF^{V600E}$	3.68	1.44	0.18	26.30	0.22	451080.00	3.70	1.07	0.17
H1975 $EGFR^{L858R,T790M}$ HGF+	1549.80	0.77	4033.50	14.89	0.24	285.48	110.87	0.36	171470.00
H1975 $EGFR^{L858R,T790M}$ $BRAF^{V600E}$ HGF+	4.59	1.88	1.02	35.76	0.23	34108.00	411.18	0.52	140300.00

Table 8: Differential equation parameters derived using Equation (S12), corresponding to experimentally derived dose response curves of afatinib in combination with either 0.5 μM crizotinib, 0.5 μM trametinib or 5 μM vemurafenib for parental and engineered 11-18 $EGFR^{L858R}$-positive lung adenocarcinoma cells.

References

[1] Eigen M, McCaskill J, Schuster P (1989) The molecular quasi-species. *Adv. Chem. Phys* 75:149–263.

[2] Hill AV (1910) The possible effects of the aggregation of the molecules of haemoglobin on its dissociation curves. *J Physiol (Lond)* 40:4–7.

[3] Chou TC, Talalay P (1984) Quantitative analysis of dose-effect relationships: the combined effects of multiple drugs or enzyme inhibitors. *Advances in enzyme regulation* 22:27–55.
